# Exploring Intrauterine Insemination in Dehydroepiandrosterone (DHEA)-Treated Women by a Clinical Study on Oocyte Quality and Pregnancy Rates

**DOI:** 10.7759/cureus.83045

**Published:** 2025-04-26

**Authors:** Fatima Bibi, Shanza Ghaffar, Tehmina Aziz, Soobia Pathan, Marvi Memon, Erum Gul, Ome Kulsoom, Aneesa Khalid, Muhmmad Hussain Shah

**Affiliations:** 1 Obstetrics and Gynecology, Ziauddin University, Karachi, PAK; 2 Obstetrics and Gynecology, Sir Ganga Ram Hospital, Lahore, PAK; 3 Obstetrics and Gynecology, Al Yamamah Hospital, Riyadh, SAU; 4 Pharmacology and Therapeutics, Liaquat Institute of Medical and Health Sciences (LIMHS), Thatta, PAK; 5 Molecular Pathology and Genetics, University of the Punjab, Lahore, PAK; 6 Pathology, Dow University of Health Sciences, Dow International Medical College, Karachi, PAK

**Keywords:** amh, dhea, fertility, fsh, intrauterine insemination, oocyte quality, ovarian reserve, pregnancy outcomes

## Abstract

Background: The administration of dehydroepiandrosterone (DHEA) has been proposed as a potential intervention for improving ovarian response among women who have diminished ovarian reserve (DOR). This study aimed to explore the associations between DHEA supplementation and the ovarian response markers in a single-arm cohort of women with DOR.

Methods: Eighty women between 25 and 40 years old with low ovarian reserve participated in this study. They received DHEA at a daily dose of 75 mg for 12 weeks before intrauterine insemination (IUI) procedures from February to August 2024. Anti-Müllerian hormone (AMH) and follicle-stimulating hormone (FSH) levels were measured in blood serum while also examining follicle appearance (≥17 mm) and endometrial layer measurements, together with pregnancy success rates. A statistical evaluation consisted of t-tests, together with correlation coefficients and logistic regression tests.

Results: Subjects who received DHEA treatment experienced both increased AMH levels (p < 0.01) and decreased FSH levels (p = 0.03). The percentage of mature follicles rose from 54.3% to 71.6% (p < 0.01). The clinical pregnancy success rate was 22 (27.5%), and there existed a significant positive relationship between enhanced follicle quality and pregnancy achievements (r = 0.41, p = 0.005).

Conclusion: DHEA supplementation was associated with improved hormonal markers, follicular development, and pregnancy rates in women with DOR undergoing IUI. The research shows that DHEA represents an effective complementary therapy for women with ovarian reserve reduction who want help with fertility. However, the lack of a control group limited the efficacy of the results.

## Introduction

The reproductive medicine field employs dehydroepiandrosterone (DHEA) as an important supportive treatment for women who have reduced ovarian reserve because this condition impairs fertility, along with pregnancy potential during intrauterine insemination (IUI) procedures [1]. Follow-up surgical procedures become challenging for women with diminished ovarian reserve (DOR), given their weak oocytes and minimal antral follicles combined with poor hormone behavior [2]. Although there is increased scientific inquiry about DHEA's reproductive effects, researchers still need to clarify how this hormone impacts reproductive outcomes [3].

Research demonstrates how DHEA can boost ovarian function via its mechanism to control androgen and estrogen levels and its positive impact on folliculogenesis and mitochondrial cell processes in oocytes [4]. Scientific research has discovered that the use of DHEA helps increase endometrial receptivity while improving the ovarian microenvironment through its stress-reducing effects and maintenance of hormonal stability [5,6]. The biological processes resulting from DHEA administration lead to better oocyte morphology together with probable higher implantation and pregnancy success during fertility treatment protocols [7]. DHEA impact assessment for clinical IUI outcomes creates potential value for individualized fertility treatment approaches [8].

This research investigation examined how DHEA affects oocyte quality alongside pregnancy rates in intrauterine insemination (IUI) cycles in order to establish precise clinical knowledge and proven methods for improving fertility outcomes within women with ovarian reserve deterioration. The researchers conducted this clinical study to determine how DHEA affects the quality of oocytes and pregnancy results for women participating in intrauterine insemination (IUI).

## Materials and methods

This single-arm prospective clinical study (reference number: 1425) assessed DOR patients aged 25-40 years who underwent intrauterine insemination (IUI) from February to August 2024, with a participant count of 80 women under reference 23/2025MS at two fertility clinics. All recruited women who attended both fertility clinics and outpatient gynecological services gave their consent to participate before study enrollment. There was no control group taken for the study due to ethical considerations related to withholding treatment from patients undergoing fertility care.

Inclusion criteria included women with confirmed DOR having serum anti-Müllerian hormone (AMH) < 1.1 ng/mL, antral follicle count (AFC) ≤ 5, regular menstrual cycles (25-35 days), and no ovarian stimulation within the last three months. Women who experienced endocrine disorders alongside autoimmune diseases and received medications that altered ovarian function were ineligible for participation in the study. The study excluded both pregnant individuals and nursing mothers. Participating women took 75 mg of oral DHEA medication each day for 12 weeks before IUI. The research team collected demographic information along with clinical information about age, body mass index (BMI), and reproductive factors, together with hormonal measurement results. Follicular monitoring was done using the transvaginal ultrasound (GE Voluson™ E10, GE Healthcare, Chicago, IL) that assessed the dominant follicle size and endometrial thickness. Follicles were considered mature if they showed a size of ≥17 mm in diameter on the transvaginal ultrasound that was assessed during the late follicular phase (cycle days 10-14). The thickness of the endometrium was measured on the day of the hCG trigger.

Researchers obtained blood samples at two time points for analyzing AMH and follicle-stimulating hormone (FSH) and estradiol results using chemiluminescence immunoassay (CLIA) (Beckman Coulter Access 2 analyzer). The researchers collected information about follicle morphology at retrieval while measuring endometrial thickness on the IUI date. Ovulation induction was performed using letrozole and gonadotropins as per clinic protocols. hCG was administered once at least one follicle reached 18 mm, followed by insemination 36 hours later using processed semen prepared by the swim-up method. The outcomes of pregnancies underwent evaluation through β-hCG measurements combined with six-week post-insemination transvaginal ultrasound examinations.

Data were analyzed using IBM SPSS Statistics for Windows version 27.0 (IBM Corp., Armonk, NY). Continuous variables were expressed as mean ± standard deviation (SD) and were compared using the paired t-test for pre- and post-DHEA comparisons. Between-group comparisons (pregnant versus non-pregnant) were performed using the independent t-test. Correlations between the variables were assessed using the Pearson correlation coefficients. A binary logistic regression was performed to evaluate the predictors of pregnancy, including post-DHEA AMH, mature follicle percentage, and endometrial thickness. A p-value of <0.05 was taken as statistically significant.

## Results

Eighty women aged 25-40 years received dehydroepiandrosterone (DHEA) therapy for 12 weeks before undergoing intrauterine insemination (IUI). The recruited women showed signs of diminished ovarian reserve according to the study criteria. On average, the participants were 31.8 ± 4.6 years old. Table 1 displays the clinical and demographic characteristics of the participants.

**Table 1:** Baseline Demographic and Clinical Characteristics SD: standard deviation, BMI: body mass index, AMH: anti-Müllerian hormone, DHEA: dehydroepiandrosterone, AFC: antral follicle count

Variable	Mean ± SD	Range
Age (years)	31.8 ± 4.6	25-40
BMI (kg/m²)	24.3 ± 3.1	19.5-30.2
AMH (ng/mL) (pre-DHEA)	0.72 ± 0.21	0.3-1.1
AFC	3.8 ± 1.2	2-5

Laboratory results for both AMH and AFC showed that the study population had established reduced ovarian reserve capacity. The researchers used restricted age ranges in combination with AFC values to create a standardized research group. The laboratory values obtained before treatment served as reference points for the evaluation of DHEA treatment results, as shown in Table 2.

**Table 2:** Hormonal Profile and Oocyte Characteristics Pre- and Post-DHEA DHEA: dehydroepiandrosterone, SD: standard deviation, AMH: anti-Müllerian hormone, FSH: follicle-stimulating hormone

Parameter	Pre-DHEA (mean ± SD)	Post-DHEA (mean ± SD)	p-value
AMH (ng/mL)	0.72 ± 0.21	1.12 ± 0.35	<0.01
FSH (mIU/mL)	12.4 ± 2.9	9.8 ± 2.5	0.03
Estradiol (pg/mL)	52.6 ± 15.4	78.1 ± 18.7	<0.01
Mature follicles (%)	54.3 ± 10.2	71.6 ± 12.5	<0.01

A significant increase in the serum AMH (p < 0.01) and a decrease in the FSH (p = 0.03) were observed following the 12-week supplementation of DHEA. Estradiol levels also elevated significantly (p < 0.01), indicating improved follicular activity. The percentage of mature follicles increased from 54.3% to 71.6% post-treatment (p < 0.01), which suggested enhanced ovarian response. The maturation of follicles showed significant advancement since patients received DHEA supplementation (p < 0.01), as shown in Table 3.

**Table 3:** Predictors of Pregnancy Outcomes After DHEA and IUI DHEA: dehydroepiandrosterone, IUI: intrauterine insemination, AMH: anti-Müllerian hormone

Variable	Pregnant group (n = 22)	Non-pregnant group (n = 58)	p-value
Post-DHEA AMH (ng/mL)	1.28 ± 0.29	1.03 ± 0.31	0.01
Mature follicles (%)	76.2 ± 8.3	68.3 ± 10.5	0.02
Endometrial thickness (mm)	9.4 ± 1.2	8.6 ± 1.5	0.04

Airnomeglicide hormone levels and mature oocyte counts rose notably among women who got pregnant through IUI procedures. The endometrial tissue expanded to a greater thickness, which supports the implantation of the embryo. The statistical data through p-values demonstrates that the chosen predictors strongly correlate with pregnancy success outcomes.

## Discussion

The research investigated how DHEA administration influenced follicle fertility quality and pregnancy success rates of women receiving intrauterine insemination for diminished ovarian reserve diagnosis. The research indicated that DHEA supplementation was linked with improved ovarian response markers, such as elevated AMH levels and better outcomes for follicle maturation. The research outcomes matched with previous studies, which found that DHEA administration improves ovarian reserve measurements and follicle maturation capacity in women who have DOR [9]. The increase in AMH and estradiol demonstrates that DHEA helps enhance ovarian responsiveness for successful fertilization and embryo implantation [10].

Better pregnancy outcomes were observed following DHEA treatment; however, due to the lack of a control group, it cannot be determined whether these outcomes are directly attributable to the treatment. Women who achieved higher mature follicle rates and attained higher AMH levels after DHEA consumption achieved significantly better pregnancy outcomes through IUI [11,12]. The data support previous research that demonstrates that better success outcomes for ART procedures (including IUI and in vitro fertilization) become possible when follicle quality improves [13]. The research results indicated that DHEA supplementation might enhance follicle quality to enhance pregnancy potential. The associations between DHEA supplementation and follicle quality do not prove direct cause-and-effect relationships. Endometrial thickness appeared as an important factor that predicted pregnancy success, together with hormonal markers [14]. A thick endometrium forms a fundamental base for implantation because its dimensions directly affect the success rates in pregnancy [15]. Future research needs to study both how DHEA works at the molecular level for positive effects and determine the best dosage amounts for distinct patient demographics.

Research findings confirm previous studies, which show that DHEA supplements boost ovarian reserve capabilities while raising both AMH and estradiol concentrations and improving follicle maturation effectiveness in women who have diminished ovarian reserve (DOR). Multiple clinical studies prove that superior follicle quality enhances the rates of successful pregnancy through IUI and IVF-assisted reproductive procedures [16]. The present study demonstrates equivalent endometrial thickness findings that confirm what previous literature establishes as fundamental to implantation success, along with successful pregnancy potential. The lack of a control group in this clinical DHEA study prevented researchers from evaluating its true benefits for treating DOR patients.

Some limitations exist within the study despite its noted strengths. Results obtained from this research might not be widely applicable because it used observational methods with a limited sample of participants. The brief monitoring period in this study fails to provide comprehensive knowledge about the sustained outcomes related to DHEA supplementation. Researchers lack precision due to the lack of molecular tests that explain how DHEA maximizes reproductive outcomes for specific patients.

## Conclusions

DHEA treatment was associated with improved ovarian response markers in women with diminished ovarian reserve (DOR) because it boosts both AMH and estradiol concentrations in the body. Effective ovarian responsiveness, along with proper follicle maturation, depends on these hormonal markers because they are vital elements for successful fertilization and pregnancy execution. This study observed that there was improved follicle quality and pregnancy rates following DHEA supplementation. However, causality could not be established due to the study design.

The treatment holds special importance for women with DOR since they encounter difficulty conceiving because their follicles show poor quality and their ovarian reserve is low. The research findings show that better follicle quality leads to improved pregnancy rates while using DHEA therapy in assisted reproductive technologies (ART). The pregnancy results of women who achieved more mature follicles showed significant improvement because follicle quality, together with uterine conditions, play an essential role in implantation.
